# Molecular characterization and exclusion of porcine *GUSB *as a candidate gene for congenital hernia inguinalis/scrotalis

**DOI:** 10.1186/1746-6148-2-14

**Published:** 2006-04-28

**Authors:** Julia Beck, Kirsten Bornemann-Kolatzki, Christoph Knorr, Helge Taeubert, Bertram Brenig

**Affiliations:** 1 Institute of Veterinary Medicine, Georg-August-University Goettingen, Burckhardtweg 2, 37077 Goettingen, Germany; 2 Institute of Animal and Genetics, Georg-August-University Goettingen, Albrecht-Thaer-Weg 3, 37075 Goettingen, Germany

## Abstract

**Background:**

Inguinal hernias are usually caused by a congenital defect, which occurs as a weakness of the inguinal canal. Porcine β-glucuronidase gene (*GUSB*) was chosen as functional candidate gene because of its involvement in degradation of hyaluronan within *gubernacular *tissue during descent of testes. Since a genome-wide linkage analysis approach has shown evidence that two regions on porcine chromosome 3 (SSC 3) are involved in the inheritance of hernia inguinalis/scrotalis in German pig breeds, *GUSB *also attained status as a positional candidate gene by its localization within a hernia-associated chromosomal region.

**Results:**

A contig spanning 17,157 bp, which contains the entire *GUSB*, was assembled. Comparative sequence analyses were conducted for the *GUSB *gene locus. Single nucleotide polymorphisms (SNPs) located within the coding region of *GUSB *were genotyped in 512 animals. Results of transmission disequilibrium test (TDT) for two out of a total of five detected SNPs gave no significant association with the outcome of hernia in pigs.

**Conclusion:**

On the basis of our studies we are able to exclude the two analyzed SNPs within the porcine *GUSB *gene as causative for the transmission of inguinal hernia.

## Background

Inguinal and scrotal hernias are usually caused by a congenital defect, which occurs as a weakness of the inguinal canal. About 1 % of all litters in German pig breeds are affected [1]. In several studies the estimated values of heritability for inguinal hernias in pigs range from h^2 ^= 0.2 [2] to h^2 ^> 0.6 [3,4]. The inguinal/scrotal hernia condition in pigs and in humans is affected by both environmental and genetic factors, with the involvement of multiple genes and likely incomplete penetrance, although the mode of inheritance has not been clarified so far and is still controversially discussed in literature [3,5-7]. In humans a high segregation ratio suggesting an autosomal dominant inheritance with incomplete penetrance and sex influence was reported. Additionally, the authors of this study noted a preferential paternal transmission of the disease gene and suggested a possible role of genomic imprinting in the aetiology of the hernia condition in man [8]. Anatomical factors such as an abnormal wide inguinal canal or a not obliterated *processus vaginalis *are regarded as predisposing for inguinal and scrotal hernia. Thus a predisposition takes place during and shortly after testicular descent. The *gubernaculum *or *genitofemoral ligament *is emerging as the key anatomical structure in the control of testicular descent. The first phase of testicular migration [9] is characterized by rapid growth of the *gubernaculum*, which serves to dilate the inguinal canal prior to descent of the testes. This is accomplished through a rapid swelling of the *gubernaculum *due to formation of hydrated space, mediated mainly by the formation and deposition of hyaluronan (HA) within the *gubernacular *tissue [10,11].

During the second phase of testicular descent the *gubernaculum *involutes, presumably due to the removal of HA, thus allowing the testes to descend into the scrotum [12]. Enzymes that act in the biodegradation of HA feature the second phase of testicular descent, these are hyaluronidase, β-hexosaminidase and β-glucuronidase.

Our hypothesis is that the swelling of the *gubernaculum *exceeds through accumulation of HA due to a diminished degradation. The inguinal ring is thus extended in an unphysiological way and may remain open predisposing the male pigs for inguinal and scrotal hernia. The limitation of HA degradation may be based on mutations in one or several of the genes coding for the HA degrading enzymes.

A genome-wide linkage scan was conducted to identify chromosomal regions involved in the inheritance of hernia in pigs [13,14]. *GUSB *first considered as functional candidate was mapped within a region on porcine chromosome 3 (SSC3) [15] that was found to be associated with congenital inguinal and scrotal hernia. Therefore, the gene was considered as a positional candidate in addition to its functional relevance.

Here we report the isolation and complete genomic characterization of the porcine *GUSB *gene, its status as a positional candidate gene for congenital inguinal and scrotal hernia in combination with results obtained from linkage and association analysis of SSC3. Further, an association study of two SNPs within the *GUSB *coding sequence was conducted to elucidate their relevance for the phenotype hernia inguinalis/scrotalis.

## Results and discussion

### Linkage and association analysis on SSC3

Linkage analysis on SSC3 was conducted using 13 microsatellites. Table 1 shows the 13 microsatellite markers their position on the chromosome and the location of the significant nonparametric LOD scores (NPL) obtained in single- and multipoint analyses. The information content was 0.62 on average. Two regions on chromosome 3 (region I: between 12.4 cM and 19 cM; region II between 98.2 cM and 102.2 cM) showed significant NPL. The singlepoint analysis revealed a significant NPL for markers SW72 (region I, p = 0.0125) and S0002 (region II, p = 0.0119), while multipoint linkage analysis gave significant NPL values for the five region I markers SW2021 (p = 0.0074), SW2429 (p = 0.0177), SW833 (p = 0.0137), SW72 (p = 0.0022), SE51018 (p = 0.0057), and for the two region II markers SW2408 (p = 0.0318) and S0002 (p = 0.0097).

The transmission disequilibrium test (TDT) was applied to all markers on SSC3 the resulting p-values are given in Table 1. TDT confirmed the results of the nonparametric linkage analysis within region I for the markers SW2429, SW833, SW72 (p ≤ 0.05) and within region II for marker S0002 (p ≤ 0.1). A significant TDT score (p ≤ 0.1) was also found for marker SW1327 located at position 109.6 cM, about 6 cM distal of region II.

### Physical distances between GUSB and microsatellite markers

GUSB was mapped by analyses of a whole-genome radiation hybrid panel [16] and fluorescence *in situ *hybridization, the most significantly linked markers were SW251 and SW72 [15]. Twopoint analyses gave a distance of 61 cR to SW72 and one of 54 cR to SW251. Hawken and colleagues estimated a conversion ratio (kb to cR) for the panel of 50.4 kb/cR on SSC3 [17]. Therefore, the distances in cR correspond to a distance of 3.07 Mb between *GUSB *locus and SW72 and one of 2.72 Mb between *GUSB *locus and SW251 at 42.3 cM [18].

These results confirmed *GUSB *as a positional candidate gene for congenital inguinal and scrotal hernia in addition to its previously proposed functional relevance.

### Structural characterization of clone TAIGP714N09100Q

Pulsed field electrophoresis after *Not*I digestion of the isolated PAC clone TAIGP714N09100Q revealed an insert size of 80 kb. Subsequent partial sequence characterization confirmed that the clone contained the whole porcine functional *GUSB *gene as well as at least one exon of the so far uncharacterized porcine *VKORC1L1 *(vitamin-K epoxide reductase paralog) gene.

### Characterization of genomic sequence and comparative sequence analyses

A contig spanning 17,157 bp, which contained the entire *GUSB *gene and 2.7 kb of its 5' and 3' flanking regions was assembled [GenBank:DQ095863] by a combination of subcloning, primerwalking and sequencing of long range PCR-products. The porcine *GUSB *gene spans over 14,436 bp (from start to stop codon) and consists of 12 exons ranging in length from 85 bp to 213 bp, which are separated by introns ranging in length from 84 bp to 3,928 bp. While the length of the exons is highly conserved between humans and pig, the size of the introns varies considerably, thus accounting for the larger size (21,521 bp) of the human *GUSB *compared to its porcine ortholog.

A comparison of the porcine *GUSB *protein-encoding region (1,959 bp) with mammalian orthologs revealed nucleotide sequence identities of 88% with the feline *GUSB *[GenBank:NM_001009310], 87% with the canine *GUSB *[GenBank:NM_001003191], 84% with the human *GUSB *[GenBank:NM_000181], and 81% with the murine *Gusb *[GenBank:J02836]. The observed identities between the known mammalian *GUSB *coding sequences are consistent with the molecular phylogenetic relationships between the different species. PiPMaker program [19] was further used to align the porcine sequence DQ095836 to a human BAC clone [GenBank:AC073261] on chromosome 7, that contains the human *GUSB *gene. Based on this alignment (data not shown) a region of 25 kb from position 35,000 to position 60,000 of the human BAC clone was used for further comparative sequence analyses. The murine sequence was taken from GenBank entry J02836. The results of screening for CpG islands and repetitive elements are shown in Table 2. DQ095863 reveals a high GC content of 54.7% compared to the mean GC content recently estimated at 49.6% and 40.7% for porcine coding sequences and introns respectively [20]. The high GC content is consistent with the GC content of 51.8% of the human *GUSB *gene. Contrary to that the GC content in the murine *Gusb *sequence is below 50% although five CpG islands were detected. Like many other housekeeping genes, human, murine, and porcine *GUSB *genes contain a CpG island that spans the promoter region, exon 1, and parts of intron 1. Only this promoter-associated CpG island is present in the three species, although the human (650 bp) and the murine (327 bp) promoter-associated CpG islands are significantly shorter than their porcine counterpart (1144 bp). Moreover, the porcine sequence contains three CpG islands in total the mouse ortholog five regions, whereas human *GUSB *contains only two CpG islands. These differences in amount and length of CpG rich regions contribute to the difference in the total GC content between the human, murine and porcine sequences. The CpG islands annotated in Table 2 matched the commonly defined criteria for a CpG island: 1) regions of DNA of at least 200 bp in length; and 2) a CG content above 50% and a ratio of observed versus expected CpGs above 0.6 [21].

The human, murine and porcine *GUSB *sequences were also screened for repetitive elements using the RepeatMasker database. The total content of repetitive sequences in human *GUSB *accounts 57.6%. Contrary, the porcine *GUSB *contains only 20.2% repetitive elements. An intermediate repeat content of 34.2% was found in the murine *Gusb *gene. In all three species the vast majority among the repetitive elements are species-specific SINE repeats, with 51.7% in the human and 31.9% in the murine but only 14.7% in the porcine *GUSB *sequence. This discrepancy can be explained by the fact that pig-specific SINE repeats (PREs) [22] are less characterized than human-specific *Alus *or murine B1-B4 elements. It is, however, most likely that this discrepancy primarily reflects a true difference rather than a biased detection in the species-specific repeat content, since all human *GUSB *introns are longer than their porcine counterparts and should therefore contain more SINE repeats.

Furthermore, 97.9% of the human SINEs are *Alu *elements, which are specific for primates. Recently, the human *GUSB *gene has been described to show an extremely high percentage of *Alu *repeats compared to the human genome average of 6-12%. The authors postulated that the *Alu *elements serve a vital function that precedes pseudogene proliferation within the human genome. Therefore, the authors concluded that most of the human *GUSB *pseudogenes have arisen from involvement of *Alu-Alu *recombination [23]. As the porcine *GUSB *contains only few SINEs in its 5' region and since we found no evidence for unprocessed *GUSB *pseudogenes within the pig genome during screening of the genomic PAC library our results support their hypothesis.

### SNP analysis

After screening all of the 12 *GUSB *exons with the adjacent intron junctions a total of five SNPs located in exons 5, 6, and 7 as well as in introns 3 and 8 were discovered. At positions 1,044 (exon 6) and 1,227 (exon 7) each (numbering refers to predicted CDS) thymine is substituted by cytosine, but both substitutions do not lead to an exchange of the encoded amino acid. The transition of guanine to adenine at position 805 (exon 5), however, changes the encoded amino acid from valine to methionine. Additionally two SNPs in intron 3 (T → C) and in intron 8 (C → T) were detected, but both of them do not alter splice motifs.

The 512 animals of the half-sib pedigree were genotyped for the three SNPs in exons 5, 6, and 7. Test assays used for genotyping and resulting allele frequencies for each of the SNPs are given in Table 3. The SNP in exon 7 was excluded from further analyses, because of the uneven allele frequency distribution with a minor allele frequency of 0.0013.

### Association analysis for GUSB-SNPs

TDT was applied to the SNPs in exons 5 and 6 to test simultaneously for association and linkage with congenital inguinal hernia. TDT analyses gave p-values of 0.229 and 0.195 for the SNPs in exon 5 and exon 6, respectively. Therefore, no significant association between the allelic variants and the disease outcome was detected.

## Conclusion

All twelve exons of the porcine *GUSB *gene were screened for SNPs to evaluate a possible contribution of the gene in the inheritance of hernia. No structural mutation that significantly alters structure of the protein has been detected. In order to evaluate if so far undetected causative mutations that lie in linkage disequilibrium with one of the detected SNPs may contribute to the disease outcome a straightforward SNP-based association analyses was conducted. No association was found for the two tested SNPs. Nevertheless it is well recognized that especially for complex diseases common association analyses in which only one gene is tested at a time might reduce the chance to identify disease susceptibility genes with relatively small effect sizes [24]. Therefore, continued finemapping and combined association analysis of candidate genes within all disease-linked chromosomal regions should shed further light on the contribution of single genes to the outcome of inguinal and scrotal hernia in pigs.

## Methods

### Animals and sampling

Blood, tissue and sperm specimen were collected on pig farms selected by different German pig breeding organisations. Before sampling, farmers reported affected animals and the hernia phenotype was proven with palpation done by veterinarians. Several additional data were recorded like position of hernia (left-sided or right-sided), number of the respective litter, date of birth. Collection of affected animals including their non-affected parents is still continuing and up to now a total of 1710 animals have been collected for the DNA repository. The affected piglets are crossbreds of the founder breeds Pietrain, Large White, Hampshire, and German Landrace. For this study, 275 affected piglets and their non-affected parents (84 boars and 153 sows) were taken. The pedigree consisted therefore of 84 boar families, each of them contains at least one affected sib pair which, if available, were completed with affected half sibs. Unaffected sibs of herniated pigs have not been collected. The size of a family ranged from a minimum of four individuals (an affected sib pair plus parents) up to a complex design with 22 individuals (five affected sib pairs and eight affected half sibs including the parents) for the largest family.

### Linkage analyses

The pedigree was genotyped for 13 microsatellite markers distributed evenly across chromosome 3. Markers and the genetic distances were taken from the published MARC U.S. Department of Agriculture map [25]. Primers used for genotyping of the 13 microsatellites on SSC3 are given in Table 4. PCR was done using puReTaq Ready-To-Go PCR beads (Amersham Pharmacia Biotech, Freiburg, Germany), reactions contained 100 ng genomic DNA and 10-50 pmol of each primer. For each reaction one of the primers was end-labelled with 6-FAM, JOE, NED or HEX and analysis of PCR-fragments was performed on an ABI Prism 3100 Genetic Analyzer (Applied Biosystems, Weiterstadt, Germany). Results of fragment analysis were evaluated using Genescan version 3.7 and Genotyper version 3.6 (Applied Biosystems, Weiterstadt, Germany).

Information content of markers, singlepoint and multipoint nonparametric linkage scores (NPL) were calculated using the program package Allegro 1.0 [26]. NPL ≥ 1.96 and p-value ≤ 0.05 were considered as significant.

### TDT analyses

Transmission disequilibrium test (TDT) [27] was conducted using the program GASSOC [28]. A p-value of 0.05 was considered as significance limit.

### PAC clone isolation and characterization

Isolation of a porcine PAC clone containing the GUSB gene from the library TAIGP714Q [29] was performed as described elsewhere [15]. The insert-size of the PAC clone (TAIGP714N09100Q) was determined by cleavage with *Not*I and separation of the fragments by pulsed field gel electrophoresis (CHEF-DR II; Bio-Rad, Munich, Germany) on a 0.8% agarose gel.

### Subcloning and sequencing

The PAC clone was digested by appropriate restriction endonucleases, and the positive fragments were subcloned into pGEM-4Z (Stratagene, Heidelberg, Germany) polylinker after southern blotting and hybridization with a *GUSB *gene specific probe. Recombinant plasmids were used to transform *E. coli *XL1-Blue (Promega, Mannheim, Germany). If necessary the obtained fragments were further subcloned after enzymatic digestion. Purified plasmid DNA were bidirectionally sequenced using the ABI Prism BigDye Terminator Cycle Sequencing Kit (Applied Biosystems, Weiterstadt, Germany) and M13fw/M13rv (5'-TGTAAAACGACGGCCAGT-3'/5'-CAGGAAACAGCTATGACC-3') primers. Remaining sequence gaps were closed by either primerwalking or by subcloning and sequencing of PCR fragments after long range PCR. Primerwalking was performed using 800 ng of PAC-DNA as template. Primers used for primerwalking are specified in Table 5a. Long range PCR was conducted using the Expand 20 kb^Plus ^PCR System (Roche, Mannheim, Germany), 50 ng of PAC-DNA and 10 pmol of each primer specified in Table 5b. Cycling conditions were 92°C for 2 min as initial denaturation, 11 cycles with 92°C for 30 sec, 55°C for 30 sec and 68°C for 8 min followed by additional 21 cycles with the same profile but with a time-increment of 10 sec per cycle, final elongation was 4 min at 68°C. All fragments were purified using QIAquick PCR purification columns (QIAGEN, Hilden, Germany) and cloned in the pGEM-T Vector System (Promega, Mannheim, Germany) for sequencing and further subcloning. All sequencing reactions were analyzed using an ABI PRISM 3100 Genetic Analyzer (Applied Biosystems, Weiterstadt, Germany). Sequence data were evaluated and overlapping contigs were generated using the software Sequencher™ 4.1 (GeneCodes, Ann Arbor, USA). Contigs were generated out of at least two independent subclones and/or PCR-products. The consensus sequence is build of plus and minus strand information.

### Bioinformatical sequence analysis

BLAST searches were conducted to identify orthologous sequence regions between species [30]. Repetitive sequences were identified, masked, and annotated with RepeatMasker [31] CpG islands were detected with CpG plot [32] using a 200-bp window and a minimum length of 200 bp. The threshold values for CpG islands were CpGobs/CpGexp > 0.6 and GC > 0.5.

### PCR conditions

All applied PCR reactions were done in compliance with the following set-up unless otherwise indicated: 50 ng of template DNA, 200 μM of each dNTP, 0.4 μM of each primer, 1.5 mM MgCl2, and 1.5 U of Taq-polymerase in 1x buffer as recommended by the manufacturer (QIAGEN, Hilden, Germany) in a total reaction volume of 25 μl.

### SNP discovery

To screen the *GUSB *exons and adjacent intron sequences (Table 5c) for the existence of SNPs, a direct sequencing approach was conducted. DNA from 15 unrelated herniated piglets from the pedigree described before and from 10 unrelated artificial insemination boars of different breeds (7x Pietrain, 2x German Landrace and 1x Large White) without any known herniated progeny was used in the comparative sequencing approach. The primer pairs specified in Table 5c were generated to amplify each exon separately. PCR amplifications were done with an initial denaturation step at 94°C for 4 min, followed by 30 cycles with 30 sec at 95°C, 30 sec at the primer specific annealing temperature and 30 sec at 72°C, followed by a final elongation for 4 min at 72°C. PCR products were sequenced directly as described above. Multiple sequence comparisons to evaluate SNPs were done with Sequencher 4.1 (GeneCodes, Ann Arbor, USA).

### SNP genotyping by PCR-RFLP

For SNP genotyping PCR-RFLP tests were developed for the two SNPs located in exon 6 and 7. RFLP reactions were performed on 10 μl of PCR-sample using 1.5 U of the respective restriction enzyme (Table 3) in a total reaction volume of 20 μl. Restriction results were subsequently electrophoretically separated on 2% agarose gels.

### SNP genotyping by tetra-primer ARMS-PCR

In order to genotype the animals for the SNP located in exon 5 a tetra-primer ARMS (Amplification Refractory Mutation System) -PCR [33] was developed. Primers (sequences and combinations given in Table 5d) were designed using an internet based program [34]. PCR reactions contained 0.6 μM of primers EX5innerA and EX5outer.f, 0,4 μM of primers EX5innerG and EX5outer.r, 2.5 units QIAGEN Taq-Polymerase (QIAGEN, Hilden, Germany), 12.5 μl of FailSafe PCR 2X PreMix I (Epicentre/Biozym, Hessisch Oldendorf, Germany) in a total reaction volume of 25 μl. The primers EX5outer.f and EX5outer.r were end-labelled with 6-FAM and analysis of PCR-fragments was performed on an ABI Prism 3100 Genetic Analyzer. Results of fragment analysis were evaluated using Genotyper version 3.6 (Applied Biosystems, Weiterstadt, Germany).

## Authors' contributions

JB performed the molecular genetic analysis of *GUSB*, sequence alignment, comparative sequence analysis and wrote the manuscript. KBK carried out the genomescan. CK edited the manuscript and participated in the coordination, design of the study. HT performed the statistical analysis. BB was responsible for funding, project coordination and manuscript preparation as senior author.

## Figures and Tables

**Figure 1:**
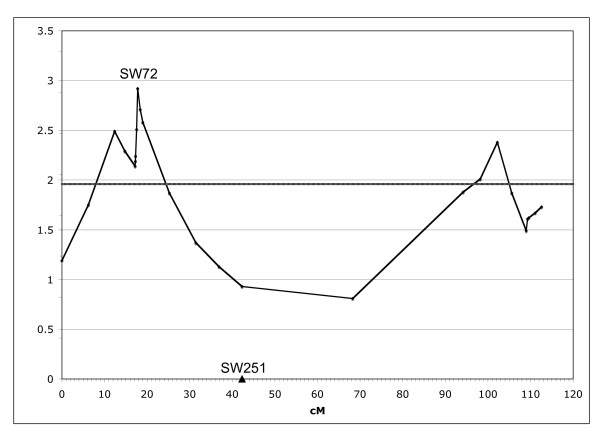
**Plot of NPL scores of multipoint linkage analysis on SSC3**. Plot of NPL scores using 13 microsatellites located on SSC3. The grey line indicates the significance limit of 1.96. With the multipoint algorithm additional points are calculated in the middle between the markers, therefore, the 26 data points shown in the graph correspond to 13 microsatellite markers tested. GUSB is localized between SW72 and SW251 as confirmed by radiation hybrid panel analyses. SW251 was not used in the genome wide linkage scan, but is indicated as rectangular on the x-axis.

**Table 1:** Results of linkage and association analyses of SSC3

Markers typed on SSC3	cM (singlepoint)	NPL in singlepoint/p-value^a^	cM (multipoint)^b^	NPL in multipoint^b^/p-value^a,b^	TDT-Score/p-value^a^
SW274	0	-0.03 / 0.5091	0 6.2	1.19 / 0.1170 1.75 / 0.0423	4.90 / 0.555
SW2021	12.4	1.370 / 0.0873	12.4 15.1	**2.49 / 0.0074** **2.29 / 0.0123**	16.55 / 0.220
SW2429	17.2	1.580 / 0.0587	17.2 17.25	**2.14 / 0.0177** **2.19 / 0.0156**	13.30 / **0.038**
SW833	17.3	0.530 / 0.2969	17.3 17.55	**2.24 / 0.0137** **2.51 / 0.0070**	11.55 / **0.021**
SW72	17.8	**2.290 / 0.0125**	17.8 30.05	**2.92 / 0.0022** **2.71 / 0.0041**	11.40 / **0.043**
SE51018	19	1.490 / 0.0694	19 25.23	**2.58 / 0.0057** 1.87 / 0.0322	9.72 / 0.373
S0701	31.46	0.460 / 0.3188	31.46 36.88	1.37 / 0.0866 1.13 / 0.1302	3.33 / 0.188
S0206	42.3	0.260 / 0.3949	42.3 68.25	0.93 / 0.1740 0.81 / 0.2075	4.69 / 0.584
SW2408	94.2	1.480 / 0.0707	94.2 98,2	1.88 / 0.0318 **2.01 / 0.0241**	9.21 / 0.602
S0002	102.2	**2.310 / 0.0119**	102.2 107.4	**2.38 / 0.0097** 1.87 / 0.0326	19.00 / **0.088**
S0397	109	0.050 / 0.4758	109 109.3	1.49 / 0.0697 1.61 / 0.0560	10.85 / 0.456
SW1327	109.6	1.730 / 0.0441	109.6 111.1	1.62 / 0.0543 1.67 / 0.0490	14.03 / **0.051**
SW349	112.6	0.380 / 0.0852	112.6	1.73 / 0.0434	13.64 / 0.136

^a ^Significant results are printed in bold letters

^b ^NPL scores were calculated using the multipoint algorithm of Allegro (version 1.0). The first value in each cell refers to the exact marker position, the second value refers to an additional theoretical position, which are set in the middle between adjacent markers.

**Table 2:** Comparative sequence statistics

	**Human**	**Pig**	**Mouse**
Cytogenetic localization	HSA7q11.21	SSC3p14-p16	MMU5G1.3
Sequence considered (bp)	25000	17121	16449
Length of gene within sequence considered (bp)	21287	14436	14148
GC content (%)	51.8	54.7	48.9
CpG islands (position within sequence considered; length (bp))	3730 – 4379; 650 11454 – 11729; 276	1510 – 2653;1144 6690 – 6910; 221 15462 – 15724; 263	2239 – 2565; 327 7084 – 7453; 370 11780 – 11982; 203 15134 – 15358; 252 15660 – 15863; 204
Repetitive sequence total (%)	57.6	20.2	34.2
SINE (%)	51.7	14.7	31.9
Alu/B1 (%)	50.6	--	9.3
B2-B4 (%)	--	--	22.1
MIR (%)	1.1	0	0
IDs (%)	--	--	0.5
LINE (%)	0.5	4.5	0
LTR (%)	4.9	0	0
Others (%)	0.5	1.0	2.3

**Table 3:** Characteristics of SNPs in *GUSB *exons

**Exon**	**Primers**^a^	**Position**^b^	**Test-Assay**	**Resulting fragments**	**Allele-Frequencies**
5	EX5outer.f/EX5outer.r/ EX5innerG/EX5innerA	805	Tetra-primer ARMS-PCR	G: 606, 342 A: 606, 308	G: 0.707 A: 0.293
6	Ex6Seq.f/Ex6Seq.r	1044	*Hpa*I-PCR-RFLP	T: 132, 109 C: 241	T: 0.651 C: 0.349
7	Ex7Seq.f/Ex7Seq.r	1227	*Hha*I-PCR-RFLP	T: 295 C: 123, 118	T: 0.987 C: 0.013

^a ^Primer sequences given in table 1

^b ^Numbering refers to CDS as outlined in GenBank:DQ095863

**Table 4:** Markers used in linkage analysis of SSC3

**Marker name**	**Sequence of forward primer (5'–3')**	**Sequence of reverse primer (5'–3')**	**T**_Ann_**(°C)**	**Alleles**
SW274	cgcacagcgacatcttttta	aagtgcagccctaaaaagaca	55	9
SW2021	gcgacacatgagataaaactgc	aatccacaggcttactcagatg	56	14
SW2429	tctttttagggtgaggatgg	catgtcccctatgaactctgtg	58	7
SW833	ctgctgttttgctgcagtg	tccactgaggtctctcactctc	58	5
SW72	atcagaacagtgcgcccgt	tttgaaaatggggtgttcc	50	6
SE51018	tctaaacaaagggcaggtgg	ggacttccatatgctgtggg	51	10
S0701	gcagagtgattcagttatac	tcatcttccctaccacc	60	3
S0206	tgggtgtggtcaacaaccaa	acgtgcctgcctctaccatc	57	7
SW2408	agacacttgtagtcgctcctcc	agacaaaaggggatgccac	57	13
S0002	gaagccaaagagacaactgc	gttctttacccactgagcca	56	13
S0397	ggctaattggagctgagaagca	agtgcagcatttccgataactctg	54	12
SW1327	agctcttgaagctcaaagacg	catacttctccaggaggaaagc	54	8
SW349	cctgttgtaggctccatgag	ctaggagtcggccctgaac	53	11

**Table 5:** Primers used for GUSB analysis

**a) Primers used for primerwalking**					
**Primer name**	**Sequence of forward primer (5'–3')**	**Strand orientation**	**Location in *GUSB***	**Template DNA**	**T**_Ann_**(°C)**

GAPIntron10.f	ggggctgagtgttagacaatgca	plus	Intron 10	PAC clone	53
GAP5prime.f	cattaacggaagattgctttggt	plus	Intron 1	PAC clone	53
3-Strich.f	gcgagagaggtactggaaact	plus	Exon12	PAC clone	50

**b) Primers used for long range PCR**					

**Primer name**	**Sequence of primer (5'–3')**	**Sequence of reverse primer (5'–3')**	**Amplified part of gene**	**Length of product**	**T**_Ann_**(°C)**

AuEx8.f/AuEx9.r	caggctgcttactacttcaa	ggtcacgaaggtcacg	Intron 8	2660	55
AuEx9.f/AuEx10.r	cgtgaccttcgtgacc	accaggagtagtaactgttca	Intron 9	1717	55
AuEx10.f/AuEx11.r	atccagagcgagtacgg	gatactgctgtagcaggc	Intron 10	3221	55
AuEx11.f/AuEx12.r	tatgtggttggagagctca	caacaggaatgctgcact	Intron 11	1843	55

**c) Primers used for SNP discovery and SNP typing**					

**Primer names**	**Sequence of forward primer (5'–3')**	**Sequence of reverse primer (5'–3')**	**Amplified Exon (Length of Exon)**	**Length of Product (bp)**	**T**_Ann_**(°C)**

GUSBEx1.f/GUSBEx1.r	atcgccttcagtcaagatgg	ctctgggccagcagttcc	Ex 1 (213 bp)	455	60
GUSBEx2.f/GUSBEx2.r	ggtgggtggcctggaatctgg	gcctcccccacccctcccta	Ex 2 (186 bp)	465	60
GUSBEx3.f/GUSBEx3.r	ttgggagggttgcctcatctct	gagctcagattacatgcctccc	Ex 3 (188 bp)	465	65.5
GUSBEx4.f/GUSBEx4.r	tgccgccagggaccattctc	ggtgccggcagagcccttct	Ex 4 (146 bp)	458	59
GUSBEx5.f/GUSBEx5.r	tcctcagagtccaggtgctcc	acctgtccggcacccctgct	Ex 5 (191 bp)	358	65
GUSBEx6.f/GUSBEx6.r	gtgaagctcacggcacagac	tgctctgagctgccctcagcc	Ex 6 (157 bp)	241	65
GUSBEx7.f/GUSBEx7.r	agcacatcttggacagggaca	gagagggagggagtcacat	Ex 7 (187 bp)	295	65
GUSBEx8.f/GUSBEx8.r	gatgtgactccctccctctc	tgaggtcccatggctggctga	Ex 8 (155 bp)	235	63
GUSBEx9.F/GUSBEx9.r	ccccaccccatcccttcctg	gggtggaaacccggctgctt	Ex 9 (89 bp)	510	59
GUSBEx10.f/GUSBEx10.r	ggtaggggtgccagcccaga	gcccaggcagggagtgaca	Ex 10 (185 bp)	430	59
GUSBEx11.f/GUSBEx11.r	gctacgtcataccttggctgtg	ccgatccgattagcttgccct	Ex 11 (140 bp)	431	65
GUSBEx12.f/GUSBEx12.r	cagcacgtctaaaccgtaagagga	actaccagattcagaccagaaact	Ex 12 (147 bp)	409	60.9

**d) Primers for tetra-primer ARMS-PCR**					

**Primer combination**	**Sequence of forward primer (5'–3')**	**Sequence of reverse primer (5'–3')**	**Length of product (bp)**	**Detected allele**	**T**_Ann_**(°C)**

EX5outer.f/EX5outer.r	gcaaccgtagctcgggacacctgaga	aagaactttcacccatcctgcccctgg	606	none	63.2
EX5innerG/EX5outer.r	ttctggatgaggaaggcagggggg	aagaactttcacccatcctgcccctgg	342	G	63.2
EX5outer.f/EX5innerA	gcaaccgtagctcgggacacctgaga	ccccccgtccccttggcaat	308	A	63.2
